# A Novel and More Efficient Oscillating Foil for Wave-Driven Unmanned Surface Vehicles

**DOI:** 10.3389/frobt.2022.759200

**Published:** 2022-03-15

**Authors:** Yan Gao, Lvcheng Xie, Tin Lun Lam

**Affiliations:** ^1^ School of Science and Engineering, The Chinese University of Hong Kong, Shenzhen, Shenzhen, China; ^2^ Research Center on Intelligent Robots, Shenzhen Institute of Artificial Intelligence and Robotics for Society, Shenzhen, China

**Keywords:** oscillating foil, asymmetric foil, WUSV, CFD simulation, speed enhancement

## Abstract

In the wave-driven unmanned surface vehicles (WUSVs), oscillating-foils are the most straightforward and widely used wave energy conversion mechanism, like the wave glider. However, WUSVs usually sail slowly compared with other types of USVs. Improving the thrust of the oscillating foil to increase its speed can help WUSVs improve their maneuverability and shorten the completion of ocean missions. This paper proposed a novel method to enhance oscillating foils’ thrust force using asymmetric cross-section shape and asymmetric oscillating motion. The thrust enhancement effect is verified by CFD simulation and pool experiment. The experimental results show that the asymmetric wing can enhance the propulsive force by at least 13.75%. The speed enhancement of WUSVs brought by this enhanced thrust is at least 7.6%, which has also been verified by simulation and sea experiment. The asymmetric foil only needs to make low-cost modifications on the traditional rigid symmetric foil to achieve the desired thrust enhancement effect.

## 1 Introduction

In recent years, marine science and exploration have developed continuously. Researchers are studying new marine environmental monitoring tools to improve their monitoring performance. At present, the mature marine monitoring tools include surface buoy, underwater buoy, shipborne monitoring equipment, etc. However, they will consume high exploration costs. Researchers prefer unmanned exploration equipment that can absorb renewable energy at sea. They can navigate independently for a long time and long range.

Compared with the limited self-carrying energy, such as gasoline and batteries, marine renewable energy has an absolute advantage in terms of endurance. The renewable energy absorbed and utilized by USVs in the ocean mainly includes solar energy, wind energy, and wave energy. Researchers have designed the following USVs using renewable energy sources: *C-Enduro* (Oh et al., 2014) is a USV that absorbs solar energy and converts it into propulsion; *Saildrone* (Mordy et al., 2017) and *Adventure Island* (Lam et al., 2016) are USVs that absorbs wind energy and converts it into propulsion; *AutoNaut* (Bowker et al., 2016) and *Wave Glider* (Hine et al., 2009) are USVs that absorb wave energy and convert it into propulsion.

The wave-driven unmanned surface vehicle (WUSV) has been widely concerned by researchers (Hine et al., 2009; Manley and Willcox, 2010; Tian et al., 2014; Johnston and Poole, 2017; Wang et al., 2020; Chen et al., 2021; Li et al., 2021; Liu et al., 2021; Lyu et al., 2021; Yiming et al., 2021), due to wave energy stability. WUSV is an unmanned vehicle that sails on the sea surface by merely utilizing wave energy as its driving power (Liu et al., 2018). Many researchers choose to use oscillating foils to absorb wave energy and convert it into the propulsion of WUSVs, as previous studies have pointed out that oscillating foils are invested as unsteady thrusters which augment ship’s overall propulsion in waves (Filippas and Belibassakis, 2014). Oscillating foils, at optimum conditions, can achieve high thrust level and efficiency, supported by extensive experimental evidence and theoretical analysis (Triantafyllou et al., 2000; von Ellenrieder et al., 2008). In WUSVs, the movement of the foils is usually passive or semi-passive, while the foils are fixed on the vehicle’s body or the separate propeller by the axis. There exist several kinds of angle limiting mechanisms to limit the maximum pitching angle of the foils. The working principle of foils is that when the vehicle rises and falls with the waves, it will generate a vertical relative flow to the foils, resulting in the foils pitching down and up, by which the foils convert the wave energy into forwarding thrust force. Compared with electric-driven USVs and wind-driven USVs, although wave-driven USVs have longer endurance and stronger survivability (for years), they have obvious sailing speed disadvantages. As a result, it may take a longer period to carry out marine surveys or monitoring tasks. This increases the time cost of users. And in some emergencies, weak mobility will make them face some inevitable survival crises. The reason for these problems is that the thrust of the oscillating foil is relatively weak. We hope to find low-cost a way to enhance the oscillating foil’s thrust to optimize the sailing performance of the WUSVs.

**FIGURE 1:**
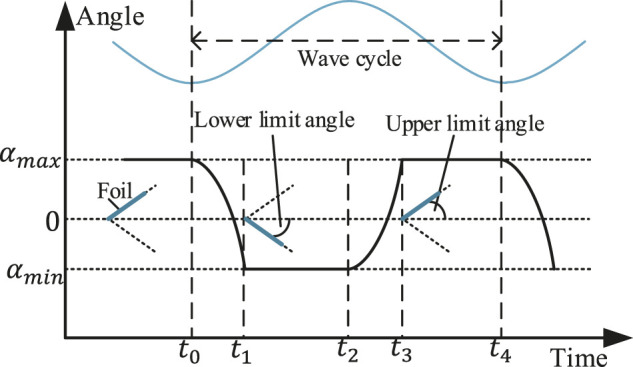
The movement of a foil in a wave cycle (*t*_0_ to *t*_4_).

As mentioned above, the working principle of foils is that when the vehicle rises and falls with the waves, it will generate a vertical relative flow to the foils. It makes the foils pitch down and up. Then foils convert the wave energy into forward propulsion force, with the foils are fixed on the body or the separate propeller of the WUSV by the axis. For foils, as shown in Figure 1, the movement in every wave cycle is composed of five steps: 1) initially at the up boundary where the angle between the foil and the horizontal is the upper limit angle and start to swing down at *t*_0_; 2) swing and reach the down boundary at time *t*_1_; 3) hold at this position until start to swing up at time *t*_2_; 4) swing and reach the up boundary at time *t*_3_; 5) hold at this position until swing down again at time *t*_4_ (Liao et al., 2016; Zhou et al., 2017; Gao et al., 2021). In some studies, the foil will be equipped with spring or torsion spring, but this paper takes the most concise passive foil as the research object.

There are three groups of governing parameters that determine the propulsion performance of the oscillating foils: environmental parameter (describing the fluid properties), geometric parameter (describing the shape of foils), and kinematic parameter (describing the motion of foils) (Wu et al., 2020). Geometric parameters are the most important and widely studied parameters because environmental parameters are uncontrollable and kinematic parameters are actually determined by geometric parameters. This means that foil’s shape is the core of its propulsion performance, which decides its thrust force in every wave cycle. One of the previous researches simulates the propulsion performance of NACA type foil numerically using the spectral difference (SD) method (Yu et al., 2013). The results indicate that relative thin foils show superior propulsion performance, on the effect of foil thickness based Reynolds numbers. Another research established a scaling law for the thrust of a foil and found that low aspect-ratio foils can improve thrust force produced by the foils when they start from rest (Lee et al., 2017). From the bionic point of view, some researchers began to study on the foil’s flexibility. One of the methods is to treat the foil as a non-extensible thin line, ignoring the foil’s thickness and shape (Zhu et al., 2014). In incompressible and laminar flows, moderate flexibility is beneficial to symmetry preservation in the wake, while excessive flexibility can trigger symmetry-breaking. In addition, one of the researches further studied the propulsion performance of bionic fin-like foils (Politis and Tsarsitalidis, 2009).

Most previous studies focused on parameters of the foil including the chord length, the maximum thickness, the span length, the aspect ratio, and some special bionic parameters. These studies are all based on the foils with symmetrical cross-section shapes, including rectangle, ellipse, and teardrop shapes. In this paper, we propose one kind of asymmetric foil with passive motion (no any actuation) used on WUSVs for the first time.

A wave glider will be used as the application carrier of our asymmetric foil technology proposed in this paper, mainly composed of float, cable, and glider (Hine et al., 2009).

- Float: The float is above the sea when it works, usually in ship form. Float’s structure needs to have a better-streamlined appearance and larger vertical cross-sectional area to reduce its forward resistance and increase its ability to respond to waves in the vertical direction. In terms of mass distribution and internal structure, it needs a lower center of gravity to ensure its stability and a larger cabin space and surface to carry the necessary equipment. A fixed fin should be set at the tail of the float to maintain stability of the float.
- Cable: The cable is composed of several wire ropes and necessary communication cables. Several wire ropes are arranged in parallel, and the direction is consistent with the longitudinal direction of float and glider. The cable should withstand the instantaneous tension caused by the great velocity potential of float under adverse sea conditions. At the connection point with float and glider, a rotation axis should be set to ensure the cable’s inclination is caused by the position difference between float and glider. And the connection point should be located in the center of gravity line of float and glider. In addition to this method, connecting gliders through triangle structures and connecting gliders through damping beams also positively affects avoiding gliders’ tilt.
- Glider: The glider is the power generation component of the whole wave glider, usually composed of the backbone, foils, tail rudder, and some sensors. The glider should follow the principle of left and right complete symmetry in structure to avoid its passive yaw force in the process of moving. The backbone should be as thin as possible on ensuring strength, and the specific shape should be adjusted according to the loaded detection equipment. Six pairs of foils were distributed on both sides of the trunk and fixed by the rotating shaft at the front of the foils. The foil’s rotation range is limited utilizing the limit bar and the C-shaped hole. According to the actual needs, springs or torsion springs can be installed to counteract the foils’ gravity at the rear end of the shaft to enhance the foils’ response to the vertical flow. There should be a tail rudder at the glider’s tail to adjust and control the wave glider’s course. Besides, a necessary IMU is needed to obtain glider heading status.

The rest of this paper is organized as follows:

Section 2: Asymmetric Foil + This section proposed an oscillating foil with an asymmetric cross-section and studied it with fluid dynamics.

Section 3: Thrust Enhancement Simulation and Experiment Verification + This section uses CFD simulation to simulate asymmetric foil’s thrust force and find better parameters to match it. Then the thrust enhancement effect is verified by a prototype and pool experiment.

Section 4: Speed Enhancement Estimation and Experiment of the Wave Glider + This section uses the kinematic model to predict the speed enhancement effect of asymmetric foil on WUSV. Finally, the practical application effect of asymmetric foil is verified by the sea sailing experiments.

Section 5: Conclusion + This section summarizes our research work and puts forward some ideas for future research.

## 2 Asymmetric Foil

For the asymmetric (shape and motion) properties of oscillating foil, researchers provide a theoretical model to predict the wake deviation of the asymmetric foil through the change of wake flow field (Chao et al., 2021, 2018). They also discussed the formation and evolution of wake structures produced by the asymmetrically oscillating foil, showing how the asymmetric oscillation affects fluid dynamics, drag-thrust transition, vortex strength, and wake jet. The research method of asymmetric oscillating foil in this paper is different from the above methods. This section and the next section will show our method of using motion decomposition to study the propulsion of oscillating foil.

In this section, our novel asymmetric foil will be proposed (Gao et al., 2021), and the theories in fluid dynamics will preliminarily prove its feasibility. A preliminary mechanical analysis is made using the panel method, point source, and point vortex theories.

### 2.1 Thrust Generation of the Foils

The foils oscillate up and down with the relative vertical water flow caused by the heaving motion of the vehicles, where the foils are fixed on the vehicles’ body or separate propeller by rotation axis. Figure 1 shows the five steps of the foils’ oscillating process in every wave cycle: 1) initially at the up boundary position where the angle between the foil and the horizontal is the upper limit angle and start to swing down at *t*_0_; 2) swing and reach the down boundary at time *t*_1_; 3) hold at this position until start to swing up at time *t*_2_; 4) swing and reach the up boundary at time *t*_3_; 5) hold at this position until swing down again at time *t*_4_.

**FIGURE 2:**
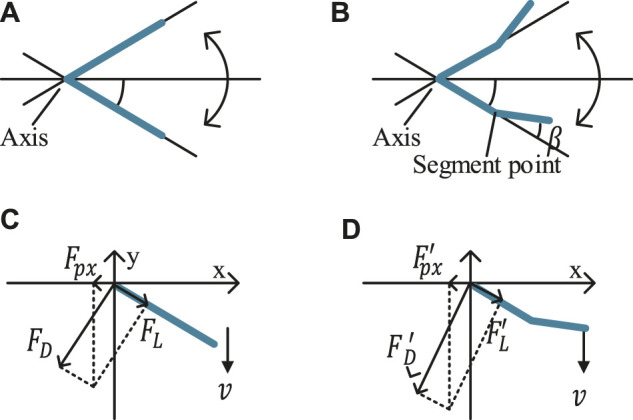
**(A,B)** Structures and movement of traditional/asymmetric foil; **(C,D)** Force analysis of traditional/asymmetric foil in time interval *T*_*l*_ [*t*_3_, *t*_4_].

In the time intervals *T*_*l*_ [*t*_1_, *t*_2_] and *T*_*u*_ [*t*_3_, *t*_4_], the force on the foil is transmitted to the WUSV through the angle limiting mechanism. How the foil responds to the water flow during these time intervals determines its propulsion performance a lot. Figure 2B shows our novel rigid asymmetric foil, with a low-cost modification on the traditional straight foil. We separate a whole foil into two parts at the segment point position and bend it at an angle *β*. The upper/lower limiting angles are the foil’s max up/down rotation angles, which determine the boundary. The initial inspiration comes from the animals’ motion. For example, the birds always bend their wings duiring flapping but not keeping straight like machine. Similarly, in WUSV applications, such bending will form an effective vortex near the segment point, and produce a high water pressure region to change the stress mode of foil during *T*_*l*_. However, there will also be thrust loss because of the asymmetric cross-section shape during *T*_*u*_. The combination of the two effects will be analyzed in the next section.

#### 2.1.1 Traditional Foil

We focus on the time interval *T*_*l*_ [*t*_3_, *t*_4_] firstly, where the force on the traditional foil and asymmetric foil is shown in Figure 2. *v* is the flow velocity relative to the foil, and *α* is the rotated angle (from the horizontal). There are form drag force *F*_*D*_ and friction drag force *F*_*L*_ on the foil, where *F*_*D*_ is perpendicular to the foil, and *F*_*L*_ has the same direction with the foil. Their magnitudes can be expressed as Eq. 1 (Pritchard and Mitchell, 2016).

$$\left\{\begin{array}{c} F_{D}=0.5\rho v^{2}S_{w}C_{D}\left(\alpha \right)\\ F_{L}=0.5\rho v^{2}S_{w}C_{L}\left(\alpha \right) \end{array}\right.$$ (1)

where *S*_*w*_ denotes the foil’s projected area with respect to *xz* plane, and *ρ* denotes the water density. *C*_*L*_(*α*) and *C*_*D*_(*α*) are the friction and form drag coefficient respectively, and they are both the functions of angle of attack *α*. We suppose that the flow is inviscid and incompressible. Here we set *C*_*L*_ as 0 and *C*_*D*_ as 2 (Haibo et al., 2011). The combined driving force *F*_*px*_ in the foil reference frame will be:

$$\begin{array}{cc} F_{px} & =-F_{D}\sin\alpha +F_{L}\cos\alpha \\ & =-\rho v^{2}S_{w}\sin\alpha \end{array}$$ (2)

#### 2.1.2 Asymmetric Foil

The above method can’t work for our asymmetric foil because the flow on it is more complex. Instead, we use the panel method to solve its driving force Anderson and Wendt (1995). We build a set of equations to get the intensity distribution, with the condition that the flow must be tangent to the foil. Through a large number of control points selected on the foil, the entire foil surface is divided into several tiny panels for force analysis. When the panel is small enough, we can assume that the intensity distribution on each panel is uniform. To reflect the interaction between the flow and the foil, we put the point sources and the point vortexes on the control points. The point source intensity and the point vortex intensity of panel *j* is *q*_*j*_ and *γ*_*j*_.

Then for panel *j*, its velocity potential of source Φ_*j*_ and vortex Ψ_*j*_ can be expressed as Eq. 3.

$$\left\{\begin{array}{cc} & \Phi_{j}=\frac{q_{j}}{2\pi }\int_{S_{i}}\ln\sqrt{{\left(x-x_{j}\right)}^{2}+{\left(y-y_{j}\right)}^{2}}\;dS_{i}\\ & \Psi_{j}=\frac{\gamma_{j}}{2\pi }\int_{S_{i}}\arctan\frac{y-y_{j}}{x-x_{j}}\;dS_{i} \end{array}\right.$$ (3)

where *S*_*j*_ denotes the area of panel *j*. Accoring to the normal velocities from both point source and point vortex velocity potential on panel *i*’s surface is 0, Eq. 4 can be built, where *v*_*∞*_ is the inflow velocity (flow at infinity) and *k* is the total number of the panel.

$$\left\{\begin{array}{cc} & v_{\infty }\frac{\partial x}{\partial n_{i}}+\overset{k}{\underset{j=1}{\sum }}\frac{\partial \Phi_{j}}{\partial n_{i}}=0,\;i=1,\ldots ,k\\ & \overset{k}{\underset{i=1}{\sum }}\frac{\partial \Phi }{\partial n_{i}}=0,\;i=1,\ldots ,k \end{array}\right.$$ (4)

The tangential velocity *v*_*Si*_ on the panel *i* is expressed in Eq. 5, with the intensity distribution.

$$v_{Si}=v_{\infty }\frac{\partial x}{\partial S_{i}}+\overset{k}{\underset{j=1}{\sum }}\frac{\partial \Phi_{j}}{\partial S_{i}}+\overset{k}{\underset{j=1}{\sum }}\frac{\partial \Psi_{j}}{\partial S_{i}}$$ (5)

Accoding to the Kutta-Joukowski condition,

$$v_{S1}=v_{Sk}$$ (6)

Then *q*_*i*_, *γ*_*i*_, and *v*_*Si*_ can be solved from Eqs 3 to 6, and pressure distribution *p*_*i*_ can be calculated using Bernoulli’s principle. The driving force *F*_*px*_ can be expressed as:

$$\;F_{px}=\overset{k}{\underset{i=1}{\sum }}\int_{S_{i}}p_{i}\cos\theta_{i}\;dS_{i}$$ (7)

where *θ*_*i*_ denotes the angle between the direction of *S*_*i*_ and the horizontal.

**FIGURE 3:**
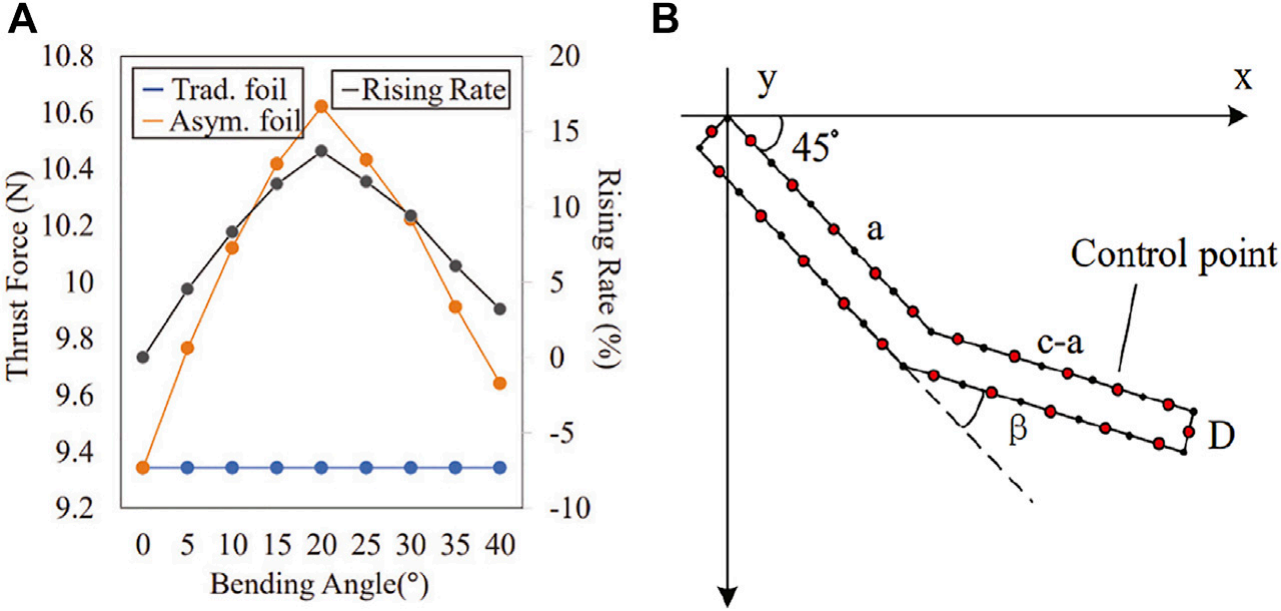
**(A)** Thrust force rising rate between traditional foil and asymmetric foil during *T*_*l*_ [*t*_3_, *t*_4_] in constant flow; **(B)** Asymmetric Foil Control Points.

Using the above numerical methods, we can preliminarily study whether our asymmetric foil has any thrust enhancement effect at a lower boundary position. It is easy to get that *F*_*px*_ of traditional foil reaches a maximum when *α* = 45° according to Eq. 2. For the asymmetric foil, we take the same value here temporarily. We divide the asymmetric foil into 10 equal parts by 11 control points on each side, shown in Figure 3B. Other parameters chosen for traditional/asymmetric foil: *v* = 5 *cm*/*s*, chord length *c* = 10 *cm*, thickness *D* = 1 *cm*, span length *L* = 100 *cm*, segment point at the center. Solving the equation (group), the traditional and asymmetric foils’ thrust force and the rising rate are shown in Figure 3A. It can be found that the thrust force of the asymmetric foil increases differently at different bending angles and reaches the maximum at 20°.

## 3 Thrust Enhancement Simulation and Pool Experiments

In the last section, we use some fluid properties to verify the feasibility of our asymmetric foil. But you have noticed that there are many ideal assumptions (such as discreting the continuous fluid) in formula derivation, which make the calculation results have a certain deviation. It is disadvantageous to use these biased results to determine the key parameters of asymmetric foil. This section will use computational fluid dynamics (CFD) simulation tools to simulate the force of foils in water flow to get a more accurate result. According to this more accurate result, the parameters of asymmetric foil will be determined and optimized in a proper order: lower limit angle, segment shape (including segment ratio and bending angle), and upper limit angle.

### 3.1 Thrust Enhancement Simulation of the Asymmetric Foil

The CFD simulation work is based on the software FLOW-3D (Flow Science, 2019). Under the same wave conditions, we will simulate and compare the thrust performance between our asymmetric foil and the traditional foil.

#### 3.1.1 CFD Simulation Setup

**FIGURE 4:**
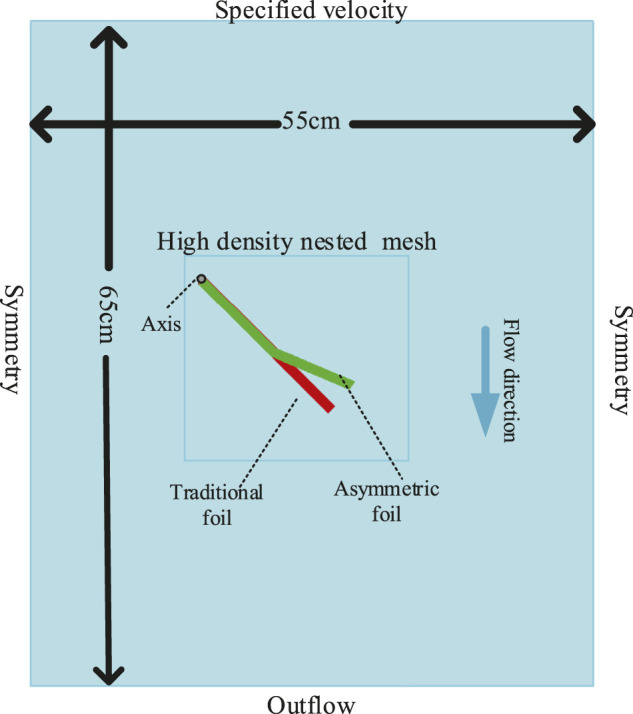
Fluid region and foils setup of the simulations. The size of the fluid region: X-55 *cm*, Y-65 *cm*, Z-100 *cm*. The boundaries of the fluid region are set as follows: XMIN-Symmetry, XMAX-Symmetry, YMIN-Outflow, YMAX-specified velocity, ZMIN-Symmetry, ZMAX-Symmetry.

As I introduced, the foils oscillate up and down with the relative vertical water flow caused by the heaving motion of the vehicles. In our simulations, traditional and asymmetric foils have the same basic size specifications: chord length 18 *cm*, thickness 1 *cm*, and span length 100 *cm*. Figure 4 shows the simulated rectangular fluid region for foils. We nested a high-density mesh in the motion region of the foils to prevent the shape-changing in the *FAVOR* rending system to keep the simulation accuracy, which is composed of 0.1 *cm* × 0.1 *cm* Cartesian grid (Ye et al., 1999). The grid number in the *Z* direction is 1 but the span length will be calculated in the pressure solver, which is a semi-3D simulation. Since we don’t know the best segment shape of our asymmetric foil in this part, we choose two parameters which seem reasonable: the segment ratio is 10/18 *cm* (The ratio of the lengths of the foil’s first section to the total foil), and the bending angle is 22°. The optimal segment shape will be found in the following parts. The position of the black spot is the foil’s rotation axis, and the upper boundary (YMAX) is the direction of fluid flowing in. The turbulence model is set as the common renormalized group (RNG) model (Yakhot and Orszag, 1986). The detailed boundaries of the fluid region are shown in Figure 4. The “Symmetry” boundary condition in FLOW-3D means that applying a zero-gradient condition at the boundary as well as a zero velocity condition normal to the boundary. As you can see, in this setting, we set the horizontal flow velocity received by foil to 0. The reason is that the horizontal speed (sailing speed) is related to the overall motion of the WUSV, and the value of this speed can not be obtained only by the CFD simulation of a foil. So we keep the forward speed consistent in all simulations, which can minimize its impact on the final simulation results.

#### 3.1.2 Optimize the Foils’ Parameters

##### 3.1.2.1 The Lower Limit Angle

In every wave cycle, the foil transmit thrust force to the WUSV during *T*_*u*_ ([*t*_1_, *t*_2_]) and *T*_*l*_ ([*t*_3_, *t*_4_]) at the boundary positions. Firstly, we need to determine the lower limit angle of the foils before we find the best segment shape of the asymmetric foil. It should be noted that the traditional foil has the same upper and lower limit angles in gravity-free condition, while the asymmetric foil doesn’t due to its asymmetric structure. The gravity-free condition means there is no gravity considered or a gravity counteracting device, like balanced NACA structure (Monnier et al., 2015) or springs. We put 40 *cm*/*s* uniform flow to the foils, which is common in the seas (Zheng et al., 2012), and the simulation time as 6 s to make sure it reaching the convergent state.

**FIGURE 5:**
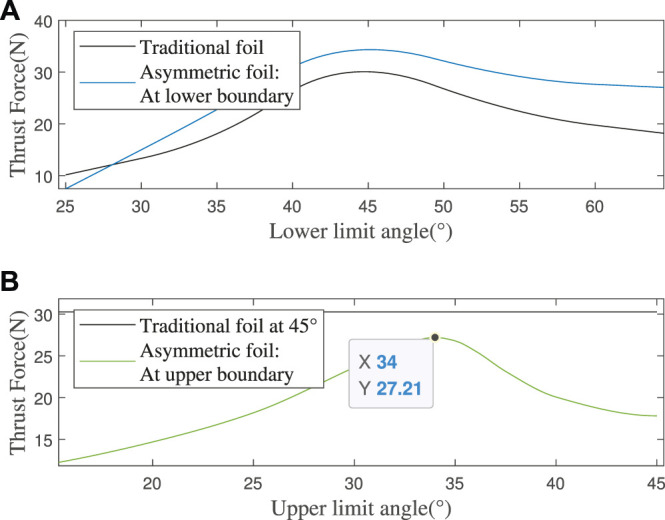
**(A)** Thrust force at different limit angles, with a constant flow speed 40 m/s **(B)** Thrust force of the asymmetric foil at different upper limit angles (less than 45°), with a constant flow speed 40 *cm*/*s*.

Through parameter scanning, we found that the best limiting angle (traditional foil’s upper and lower limit angles and asymmetric foil’s lower limit angle) to make the foils transmit the most thrust force is about 45°, as shown in Figure 5A. We don’t need an exact value of the asymmetric foil’s lower limit angle because its segment shape has not and will be optimized. What’s more, the thrust force’s change rate of the asymmetric foil is faster at the low angle and slower at the high angle than the traditional foil in the simulation interval because of its asymmetric structure.

##### 3.1.2.2 Bending Angle and Segment Ratio of Asymmetric Foil at the Lower Limit Angle

**FIGURE 6:**
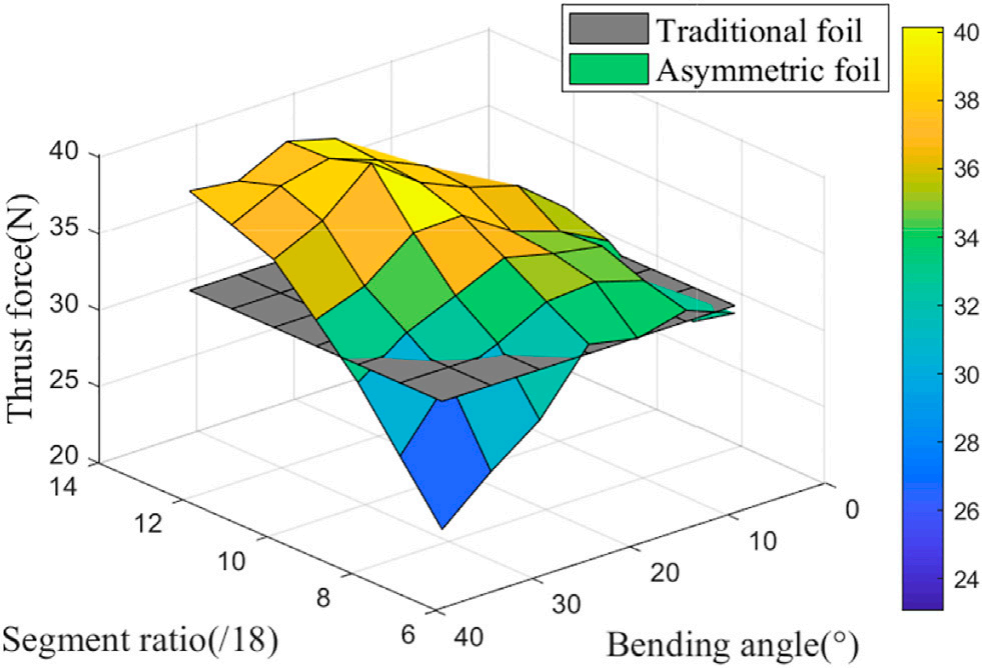
Thrust force of the asymmetric foil with different parameter pairs (segment ratio and bending angle) at the lower limit angle 45°, with a constant flow speed 40 *cm*/*s*.

The above-selected asymmetric foil’s segment shape can be optimized with this lower limit angle: segment ratio and bending angle. Through parameters scanning, the foils’ thrust force with different parameter pairs in the flow is shown in Figure 6.

The gray plane is the contrasting force of the traditional foil. We can see the asymmetric foil may perform better or worse than the traditional foil. It generates less thrust force at the low segment ratio and large bending angle (the first section of the foil is too short and the bending angle too large, like 6/18 and 35°). The optimal thrust enhancement effect can reach 31.76% ((39.87N/30.26N)-1) during *T*_*l*_ with the parameter pair: the segment ratio is 11/18, and the bending angle is 25°.

##### 3.1.2.3 The Optimal Upper Limit Angle of Asymmetric Foil

Due to the asymmetric foil’s special shape, there is always a thrust loss during *T*_*u*_, and a smaller upper limit angle (less than 45°) is needed to prevent this loss. With the same method, I get the simulation results as shown in Figure 5B. From the parameter scanning results, the foil has a minimum thrust loss of 10.08% (27.21N/30.26N-1) at an upper limit angle of 34°.

So far, we have determined all the asymmetric foil’s optimal parameters: the lower limit angle of 45°, segment ratio of 11/18, bending angle of 25°, and upper limit angle of 34°.

#### 3.1.3 Pitching Motion Simulation in Standard Wave

With the above foils’ parameters, we need to simulate their pitching motion in the wave to obtain the relationship of time intervals *T*_*l*_ and *T*_*u*_ between the traditional foil and our asymmetric foil. We selected a common wave case in the seas Zheng et al. (2012): the wave amplitude *A* of 1.2 m, and the fixed-point wave period *T*_*w*_ of 4 s. Under this wave condition, the wave function and the vertical relative flow to the foil is:

$$\left\{\begin{array}{cc} & y\left(t\right)=-0.6\cos\left(\frac{\pi t}{2}\right)\\ & v\left(t\right)=0.3\pi \sin\left(\frac{\pi t}{2}\right) \end{array}\right.$$ (8)

**FIGURE 7:**
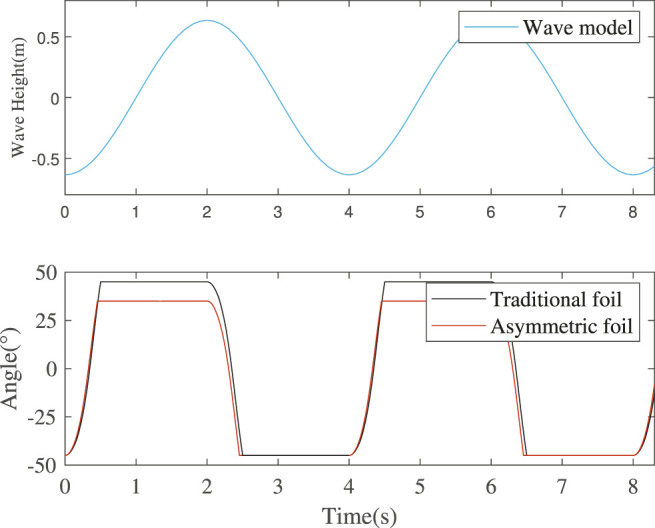
Foils’ rotation in a standard wave with amplitude 1.2 m and fixed-point wave period 4 s.

From the simulation result in Figure 7, I get the values of time intervals $T_{u}^{T}$ and $T_{l}^{T}$ of traditional foil are both 1.49 s (74.5% of half wave period), while those are 1.53 s ( $T_{u}^{A}$ ,76.5% of half wave period) and 1.54 s ( $T_{l}^{A}$ , 77% of half wave period) of asymmetric foil. Due to the asymmetric structure and smaller rotation angle (11° less) of the asymmetric foil, the time intervals of different foils follow that: $T_{u}^{A}\approx T_{l}^{A}>T_{u}^{T}=T_{l}^{T}$ .

#### 3.1.4 Thrust Enhancement Effect

In waves, the relative flow to the foil changes with time rather than a constant flow, as shown in Figure 7. To obtain the asymmetric foil’s overall thrust enhancement effect in the waves, we also need to obtain its thrust enhancement/loss effect under other flows.

**FIGURE 8:**
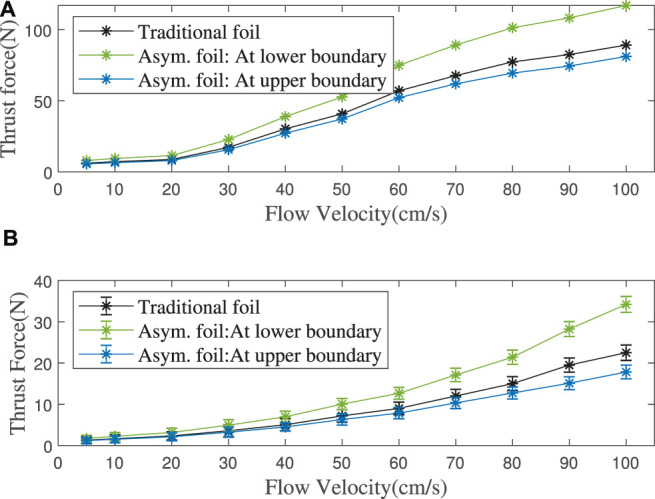
**(A)** Simulation: Foils’ thrust force in different flow velocities. **(B)** Experiment: Foils’ thrust force with different flow velocities at upper (pitching up)/lower (pitching down) limit angles, mean value of 10 experiments’ data.

**FIGURE 9:**
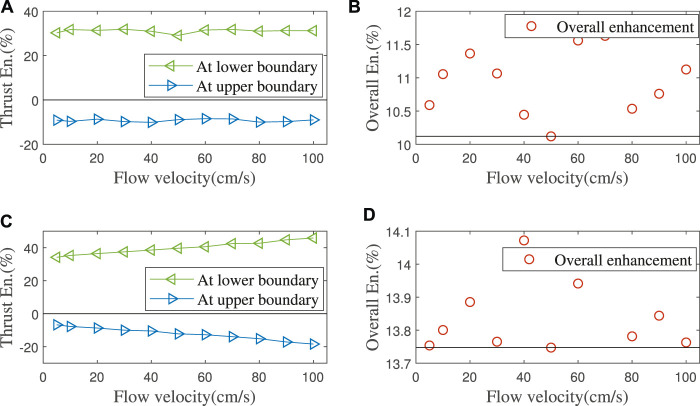
**(A,B)** Simulation: Thrust enhancement/loss percentage of the asymmetric foil at lower/upper limit angle. Overall thrust enhancement effect is calculated by Eq. 9. **(C,D)** Experiments: Thrust enhancement/loss percentage of our asymmetric foil at upper/lower limit angles and its overall thrust enhancement effect.

The asymmetric foil’s working effect is simulated in the flow velocity range of 1 m/s, and the result is shown in Figure 8A. Figures 9A,B shows the thrust enhancement/loss effect of our asymmetric foil under various flows. Using to Eq. 9, its overall thrust enhancement effect *E*_*o*_ is 10.12%. Here we assume that the flow on the foil in the wave has no relationship with the flow at the previous moment. This assumption will slightly affect the simulation accuracy. Still, it can greatly expand its universality because it can make the results cover a wider range of wave conditions than single wave simulation.

$$\begin{array}{cc} E_{o} & =\frac{\int_{T_{l}^{A}}\left(1+E_{t}\right)dt+\int_{T_{u}^{A}}\left(1+L_{t}\right)dt}{\int_{T_{l}^{T}}1dt+\int_{T_{u}^{T}}1dt}-1\\ & \geq \frac{\int_{T_{l}^{T}}\left(1+E_{t}+1+L_{T}\right)dt}{2T_{l}^{T}}-1\\ & \geq \min\frac{E_{t}+L_{t}}{2},t\in T_{l}^{T} \end{array}$$ (9)

*E*_*t*_/*L*_*t*_ are the thrust enhancement/loss percentage at time *t*. It can be verified that as long as the wave condition meets the following: $\frac{2\pi A}{T}\leq 1$ (the maximum relative flow velocity is in the range of our simulations, 1 m/*s*), this thrust enhancement of asymmetric foil is always effective.

The method of determining asymmetric foil’s key parameters is summarized into pictures and shown in Supplementary Material.

### 3.2 Glider and Pool Experiments

We carried out the hydrodynamic experiments in a water pool with our glider prototype, which could carry different foils. An experimental framework to test the thrust enhancement effect of the prototype is built.

#### 3.2.1 Experimental Setup

**FIGURE 10:**
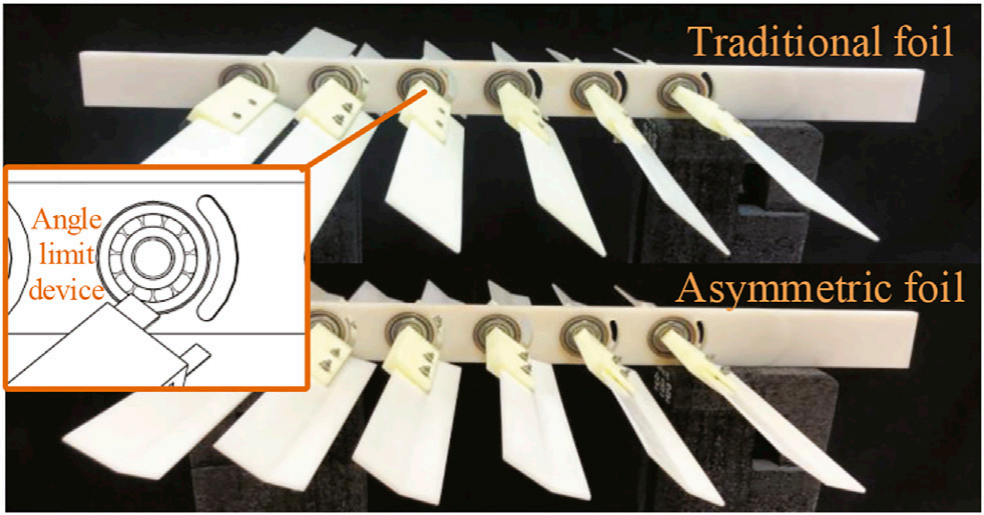
Propeller mini-prototypes made by 3D printing with density around 1 *g*/*cm* ^3^ (material density 1.25 *g*/*cm* ^3^× fill rate 80%). Upper side: propeller with the tradition foils; Lower side: propeller with our asymmetric foils.

**FIGURE 11:**
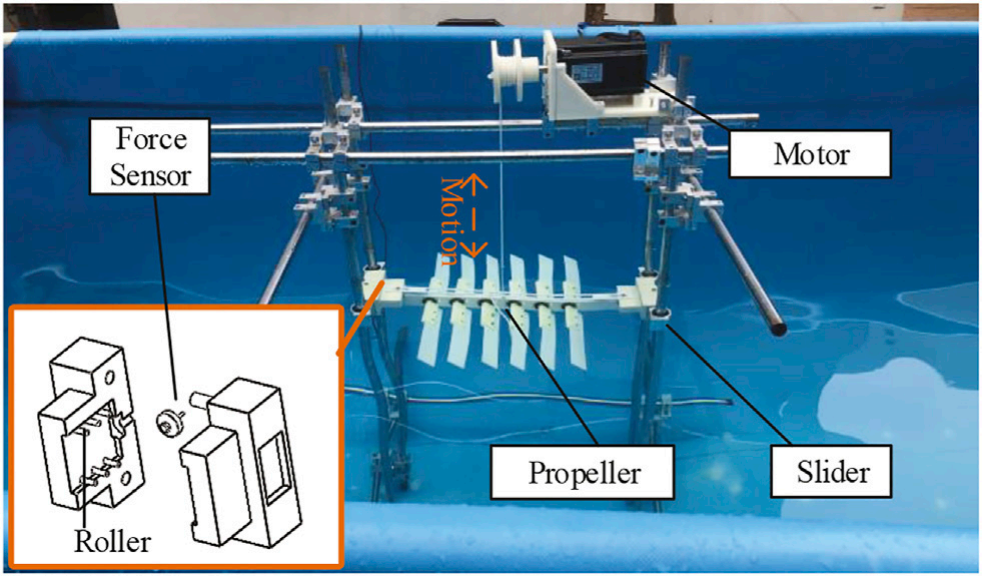
Experiment framework: two heavy slider, a pressure sensor, several rollers in side the slider; all in a water pool with the water elevation about 1.3 m.

Figure 10 shows our prototype of the glider, which is easy to install and operate. The glider is composed of six pairs of foils and one backbone. The foils (both traditional and asymmetric foils) are dismountable though the connection device. The angle limiting mechanism is implemented by the connection device and backbone with the arc holes, which are different for two kinds of foils. The full glider is 57 *cm* long and 42 *cm* wide, including the 20 *cm* long foils, with the distance between every pair of neighbor foils is 5.5 *cm*. Other parameters are chord length 4 *cm*, and thickness 0.2 *cm*. The experimental framework is shown in Figure 11. The glider and the corresponding thrust force detection device are placed in a water tank with scale: 4 m length × 2 m width × 1.5 m height. A thin rope suspends the glider on a wire wheel controlled by an electric motor. The propeller’s head and tail are inserted into the sliders equipped with a force sensor and several rollers to reduce the friction.

#### 3.2.2 Thrust Enhancement Experiments in Constant Flows

Rather than to directly create water flow like some previous researches Lua et al. (2016), we use the electric motor to pull the propeller to create the relative flow. The same as the simulation, I also set the horizontal flow as 0. For each flow velocity, I take the average thrust force of ten times experiments, as shown in Figure 8B.

There are some differences between the CFD simulation and the hydrodynamic experiment. The thrust enhancement/loss effect of asymmetric foil increases with the flow velocity, which the coupling between multiple foils may cause. According to Eq. 9, the minimum overall thrust enhancement effect *E*_*o*_ in the wave is 13.75% (*E*_*o*_ ≥ min (*E*_*t*_ − *L*_*t*_)/2 = 0.137 5), as shown in Figures 9C,D. As noticed above, it can be verified that as long as the wave condition meets the following: $\frac{2\pi A}{T}\leq 1$ (the maximum relative flow velocity is in the range of our simulations, 1 *m*/*s*), this thrust enhancement is always effective. Of course, the extremely stable sea conditions that cannot make the foils oscillate are not in this range (for all passive oscillating-foils but not only our asymmetric foils).

#### 3.2.3 Thrust Enhancement Verification in Waves

**FIGURE 12:**
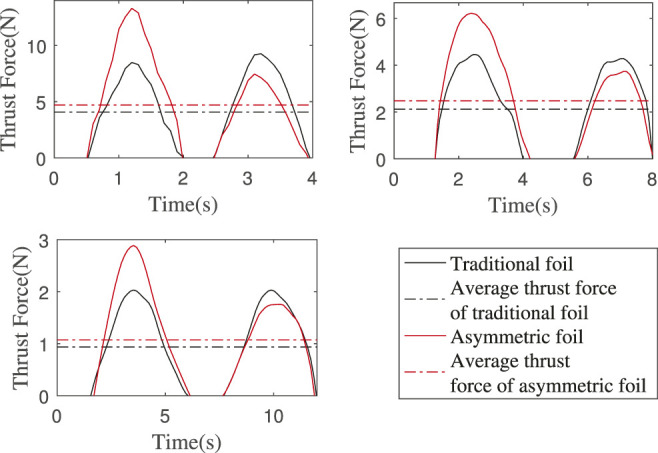
Foils’ thrust force in a complete wave period. Wave amplitude is 0.8 m and wave periods are 4, 8, and 12 s. The thrust enhancement effects: 15.59%, 16.77%, and 14.49%.

We use the motor to simulate the wave with amplitude *A* as 0.8*m* and the wave period *T*_*w*_ as 4, 8, and 12 s. Figure 12 shows the thrust force of the foils in a complete wave period. The average thrust forces of asymmetric foil and traditional foil in each wave condition are also shown, and the thrust enhancement effects are 15.59%, 16.77%, and 14.49%, all larger than the minimum *E*_*o*_ = 13.75*%*.

## 4 Speed Enhancement Estimation and Experiment of the Wave Glider

It is an economical and effective method to estimate the motion performance by establishing the numerical model of dynamics and kinematics before real sea experiments. However, for researchers, due to the lack of hydrodynamic performance parameters of asymmetric foils (or any other new type of foils), the motion simulation cannot be carried out. Our method is to combine the CFD simulation results in the previous section into the numerical model, which makes the motion simulation of WUSV equipped with new type of foils very convenient.

For marine vehicles, the most famous and widely used model is Fossen’s model (Fossen, 2011), which was derived in the form of Newton-Euler. However, the existing models, including Fossen’s model, are all aimed at monomer marine vehicles. The wave glider has a more complex two-body structure and a more complex propulsion method. Based on the Kane vector operation modeling method, a simplified dynamic model is proposed, which does not consider the hydrodynamic coupling in multiple directions, but only considers the vertical motion (Qi et al., 2013). A three-body distributed four-degree of freedom kinematic model based on the Newton-Euler approach considers the second-order wave drift force and the first-order wave force in the vertical direction and calculates the hydrodynamic parameters by using the potential flow theory and empirical formula (Wang et al., 2019). Compared with the distributed model, a centralized six-degree freedom model can succinctly and perfectly describe wave glider motion. Still, only the combination of empirical results and Rayleigh distribution is used for the thrust generated by foils (Kraus et al., 2012). This paper will make further modifications and optimization to simulate the wave glider’s motion based on this model.

### 4.1 The Wave Glider Prototype and Reference Frames

**FIGURE 13:**
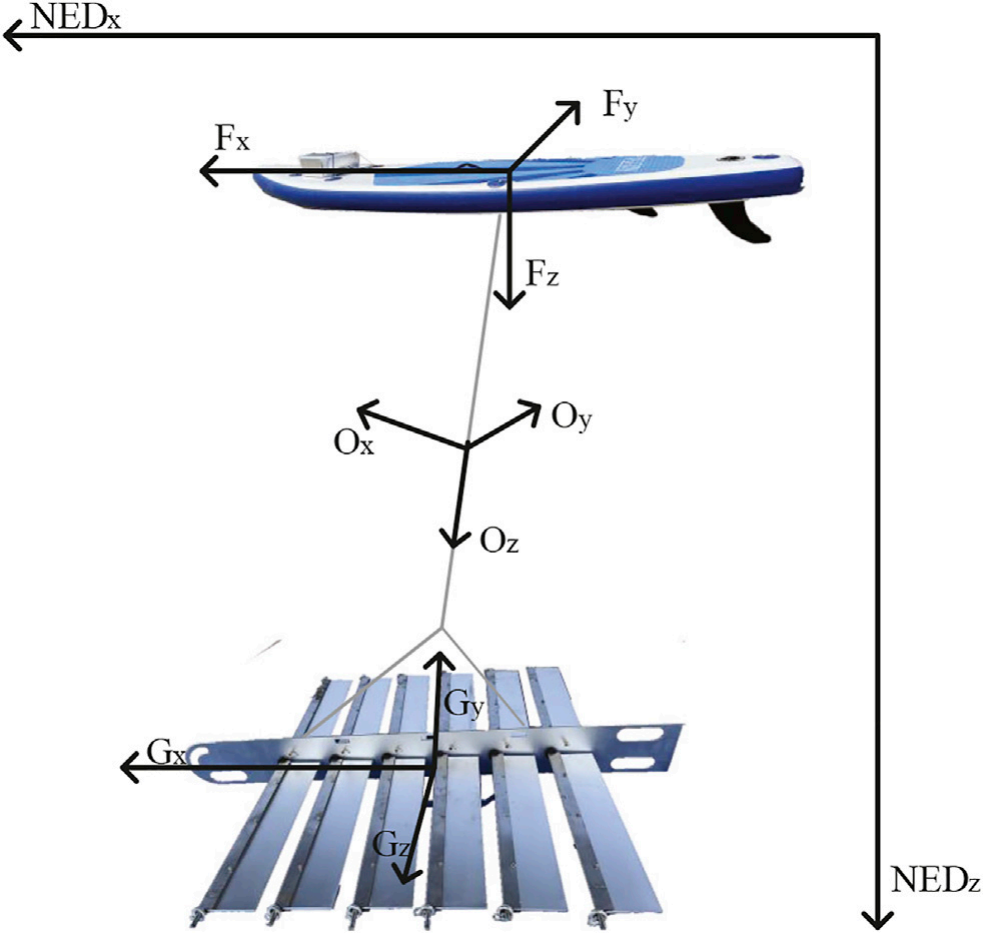
The four reference frames:Earth-fixed frame (^*NED*^); Float Frame (^*F*^); Glider frame (^*G*^); System frame (^*O*^).

Our wave glider prototype is shown in Figure 13. The total length of the glider is 140 *cm*. Two wave gliders carry traditional and asymmetric foils with 50 *cm* span length and 14 *cm* chord length, respectively. The structural parameters of asymmetric foil are set as the optimized results above. In defining the coordinate system and wave glider motion parameters, it is assumedThe structural parameters of asymmetric foil are set the same as the above simulation results. that the earth is an ideal plane, regardless of the earth’s curvature and rotation. The geodetic coordinate system is an inertial reference frame. And because the cable is always in a tight state in moving, it can be regarded as a rigid body. Due to the two-body structure and unique motion mode of the wave glider, it is necessary to establish multiple frames to describe its local and overall motion. Therefore, the establishment of four frames is also shown in Figure 13.

1. Earth-Fixed Frame (*S* ^*NED*^): The North-East-Down (NED) frame, regard as an inertial frame. *x* is positive north, and *y* is positive east.
2. Float Frame (*S* ^*F*^): This coordinate frame coincides with the principal axes of inertia of the float. The coordinate system’s origin is located at the center of gravity of the float, where *x* ^*F*^ points forward, *y* ^*F*^ points starboard, and *z* ^*F*^ points down.
3. Glider Frame (*S* ^*G*^): This coordinate frame coincides with the principal axes of inertia of the glider. The coordinate system’s origin is located at the center of gravity of the glider, where *x* ^*G*^ points forward, *y* ^*G*^ points starboard, and *z* ^*G*^ points down.
4. System Frame (*S* ^0^): The origin of the coordinate system is located at the centroid of the Wave Glider. *x* ^0^ is perpendicular to the cable and points in the forward direction. *y* ^0^ takes the direction of the cable and points to the glider from the float. The direction of *y* ^0^ conforms to the right-hand rule.

The representation of motion variables adopts the Society of Naval Architects and Marine Engineering (SNAME) format Fossen (1999). The positive direction of force and velocity points to the coordinate square axis, and the positive direction of angle and angular velocity follows the right-hand rule.

- Roll angle *ϕ*: The angle between the plane *xoz* and the plane of the plumb containing the x-axis. According to the right-hand rule, from the vertical plane to the symmetrical plane of the reference frame around the x-axis is the *ϕ*’s positive direction.
- Pitch angle *θ*: The angle between z-axis and vertical plane. *θ* is positive when the z-axis is forward.
- Yaw angle *ψ*: The angle between the y-axis and vertical plane. The positive direction of *ψ* follows the right-hand rule.

In addition, the upper right corner is marked with ’F’, ’G’ or ’0’ to determine which department of wave glider this quantity belongs to (for example, *u* ^*G*^ represents glider’s forward speed). The upper left corner mark indicates the value of the quantity converted to a certain reference frame (e.g. ^0^ *u* ^*G*^ indicates the glider’s forward speed converted to the system frame).

### 4.2 Kraus’ Model

A standard representation of a 6DOF Fossen’s model (Fossen, 1999) is:

$$M\dot{v}+C\left(v\right)v+D\left(v\right)v+g\left(\eta \right)=\tau$$ (10)

Where

- M ∈ R6×6: inertia matrix including added mass
- C(v) ∈ R6×6: matrix of Coriolis and centripetal terms
- D(v) ∈ R6×6: fluid damping matrix
- g(η) ∈ R6×1: restoring force matrix under static force (gravity)
- τ ∈ R6×1: vector of control input
- v = [u, v, w, p, q, r]T
- η = [x, y, z, ϕ, θ, ψ]T

To make Fossen’s model suitable for the wave glider, Kraus Kraus et al., 2012 made the following modifications to the pose (***η***) and velocity (**v**):

$$\begin{array}{cc} & v={\left[u^{0},\;v^{0},\;p^{0},\;q^{0},\;r^{F},\;r^{G}\right]}^{T}\\ & \eta ={\left[x^{0},\;y^{0},\;\phi^{0},\;\theta^{0},\;\psi^{F},\;\psi^{G}\right]}^{T} \end{array}$$ (11)

The full six DOF set of nonlinear dynamic equations of motion as:

m0000000m0000000Ixx0000000Ixx0000000IzzF000000IzzGu˙0v˙0p˙0q˙0r˙FFr˙GG+00000−m0v000000−m0u000000−Ixx0q000000Ixx0p0000000000000u0v0p0q0rFFX0+X0000000Y0000000K0000000M0000000NF000000NG+XHSG0−YHSG0−YHSG0|0rG0|XHSG0|0rG0|00=XThrustG0+XuδG0(δ)YThrustG0+YuδG0(δ)−(YThrustG0+YuδG0(δ))|0rG0|(XThrustG0+XuδG0(δ))|0rG0|0NuuδGG (12)

The meanings of the parameters in the model are:

- *m* ^0^: Mass of the wave glider system
- |^0^ *r*_*G*0_|(|^0^ *r*_*F*0_|): The distance between the system’s CG of and the glider’s (float’s) CG
- $I_{xx}^{0}$ : $m_{x}^{F}{|}^{0}r_{F0}{|}^{2}+m_{x}^{G}{|}^{0}r_{G0}{|}^{2}$

- X0−M0 : Damping of the wave glider system in the corresponding direction
- NF(NG) : Yawing damping of the float (glider)
- XHSG0,YHSG0 : The restoring force caused by the gravitational force
- XThrustG0,YThrustG0 : The glider’s thrust force
- XuδG0(δ),YuδG0(δ) : Damping force caused by the tail rudder changing the angle
- NuuδGG : Yaw force caused by the tail rudder changing the angle

### 4.3 Speed Enhancement Estimation

To build the above model in Matlab-Simulink, we need to solve the expression of $\dot{v}$ first (without considering the tail rudder):

u˙0=−(X0+XHSG0−XThrustG0−rGGm0v0)/m0v˙0=−(Y0+YHSG0−YThrustG0−rGGm0u0)/m0p˙0=−(K0−YHSG0|0rG0|+YThrustG0|0rG0|−rGGIxx0q0)/Ixx0q˙0=−(M0−XHSG0|0rG0|+XThrustG0|0rG0|−rGGIxx0p0)/Ixx0r˙FF=−N/IFzzFr˙GG=−N/IGzzG (13)

Using the integral module in Simulink, we can integrate $\dot{v}$ into **v** and build the whole model.

Compared with Kraus’s motion simulation, we use fewer empirical values in the simulation process. Kraus needs the historical empirical value in the foil thrust input, making this method only suitable for the motion simulation with the existing foils after sailing experiments. We can estimate the speed of the wave glider equipped with any new foils by combining the CFD simulation results in the previous section. For other parameters, most of them are set based on the prototype. However, due to the difficulty of measuring some parameters, such as the damping coefficient of the wave glider, we also set them according to the empirical value obtained by Kraus’ experiment. However, this does not affect our desired simulation results - speed enhancement effect, because these settings will be kept the same in the simulations.

In the numerical model, we input the enhanced thrust during *T*_*l*_, and the lost thrust during *T*_*u*_ obtained from the above simulation to ensure that the average thrust/wave cycle and momentum (1102 *Ns* and 1255 *Ns*) enhancement remains at 13.75%. The detailed thrust input method is in the Supplementary Material.

**FIGURE 14:**
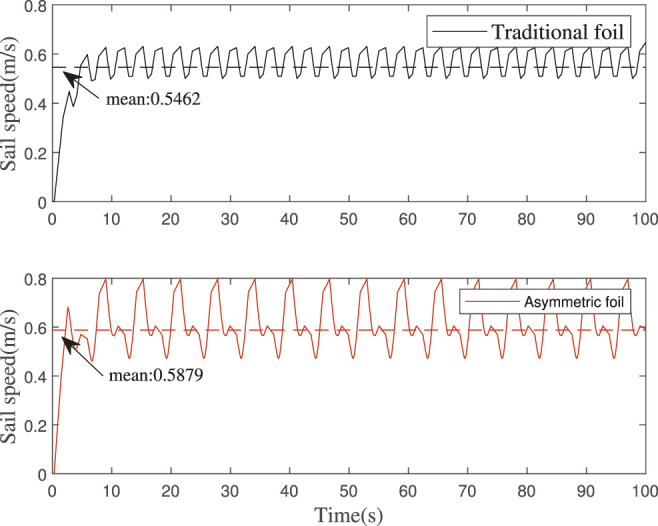
Wave glider speed simulation: The average speed of wave glider with the traditional foils is predicted to be 0.5462 m/*s*, while the average speed of wave glider with the asymmetric foils is predicted to be 0.5879 m/*s*.

The simulation results are shown in Figure 14. The average speed of wave glider with the traditional foils is predicted to be 0.5462 m/*s*, while the average speed of wave glider with the asymmetric foils is predicted to be 0.5879 m/*s*. We can get that the wave glider’s speed can be increased by at least 7.6% by using the asymmetric foils.

### 4.4 Speed Enhancement Experiment

The sea trials were carried out in the sea area of Qixing Bay in Shenzhen, China. The experimental time and marine environment at that time is that: Time: ∼3:00 UTC, 10/05/2021; Wind: ∼ 3 *kt*; Wave amplitude: 0.2–0.3 m. We put two prototypes with different foils in the same place and at the same time. The following positions of the two prototypes are somewhat different, but the distance is kept within 150 m, and the difference in wave environment is enough to be ignored.

**FIGURE 15:**
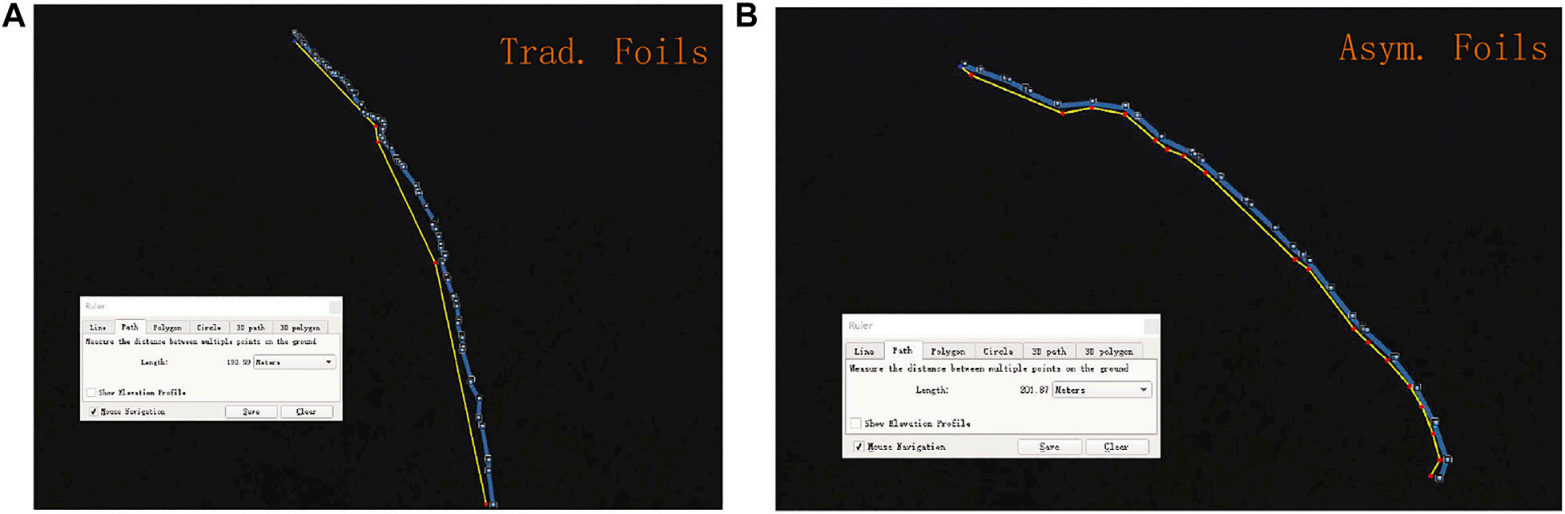
**(A)** Sailing record of the wave glider with traditional foils: From 3:08:39 to 3:37:58; The sailing distance is 190.59 m. **(B)** Sailing record of the wave glider with asymmetric foils: From 3:08:38 to 3:37:09; The sailing distance is 201.87 m.

The marine navigation record is shown in Figure 15. The average speed of wave glider with traditional foils is 190.59 m/1759 s = 0.108 4 m/*s*. The average speed of wave glider with asymmetric foils is 201.87 m/1711 s = 0.118 0 m/*s*. According to this, the wave glider’s speed increased by 8.89%, which is higher than the lowest value of 7.6% obtained from the numerical simulation. In this experiment, the asymmetric foil’s performance is beyond our expectation.

## 5 Conclusion

In this paper, a novel asymmetric foil is proposed, which only needs to make low-cost modifications on the traditional rigid symmetric foil to achieve the desired thrust enhancement effect. I focus on the advantages of our novel asymmetric foil compared with the traditional foil in WUSVs’ sailing. The combination of simulation and experiment verified that our novel asymmetric foil could provide larger thrust (at least 13.75%) to WUSVs in a broad range of wave conditions. The enhanced propulsion force is input into the wave glider’s kinematic model, and the asymmetric foil is mounted on the wave glider prototype to verify the minimum speed enhancement (at least 7.6%). This speed increase can shorten a 1-year ocean survey mission to 11 months.

### 5.1 Contributions

The main contributions of this work are as follows: a low-cost oscillating foil with asymmetric cross section and its supporting asymmetric motion mechanism are proposed to make WUSVs obtain greater propulsion and higher speed in waves; This paper presents a method to measure glider thrust and the device in the pool; a method combining CFD simulation and numerical model simulation is proposed to obtain more accurate results of thrust and motion simulation of the foil.

### 5.2 Future Work

There are some ideas and suggestions to improve the new foils’ performance and research.

- Deformable foil: As the current version of the foil can not be deformed, it will cause a thrust loss during *T*_*u*_. It is a great pity and we hope it can generate more thrust both during *T*_*u*_ and *T*_*l*_. If the foil is designed as a deformable mechanism, this goal can be achieved.
- Bionic (soft) foil: It can be found that the invalid rotation time of the foil accounts for a large part of the whole movement cycle. The reason is that the rigid foil will be subject to greater water resistance in the process of rotation. If a soft foil is used, the rotation time is expected to be greatly reduced, thus increasing the foil’s effective working time to obtain more propulsion.
- Dynamic CFD simulation: There is a certain deviation in the calculation of the thrust force of the static foil when it is subjected to the water flow, because the foil is advancing at a different forward speed, which is difficult to obtain. If the overall dynamic simulation of WUSVs can be realized, the simulation results are expected to be more accurate.

## Data Availability

The datasets presented in this study can be found in online repositories. The names of the repository/repositories and accession number(s) can be found below: https://github.com/GavinGaoYan/SeaTrialData.git.
