# Supplementary material for: A Novel and More Efficient Oscillating Foil for Wave-Driven Unmanned Surface Vehicles
Source: Front Robot AI. 2022 Mar 15;9:759200. doi: 10.3389/frobt.2022.759200 (PMC8964531; doi:10.3389/frobt.2022.759200)
Supplement: Supplementary file 1 [file DataSheet1.pdf]

Supplementary Material

1 SUPPLEMENTARY DATA

Supplementary Material should be uploaded separately on submission. Please include any supplementary data, figures and/or tables. All supplementary files are deposited to FigShare for permanent storage and receive a DOI.

Supplementary material is not typeset so please ensure that all information is clearly presented, the appropriate caption is included in the file and not in the manuscript, and that the style conforms to the rest of the article. To avoid discrepancies between the published article and the supplementary material, please do not add the title, author list, affiliations or correspondence in the supplementary files.

2 SUPPLEMENTARY TABLES AND FIGURES

For more information on Supplementary Material and for details on the different file types accepted, please see the Supplementary Material section of the Author Guidelines.

Figures, tables, and images will be published under a Creative Commons CC-BY licence and permission must be obtained for use of copyrighted material from other sources (including re-published/adapted/modified/partial figures and images from the internet). It is the responsibility of the authors to acquire the licenses, to follow any citation instructions requested by third-party rights holders, and cover any supplementary charges.

2.1 Figures

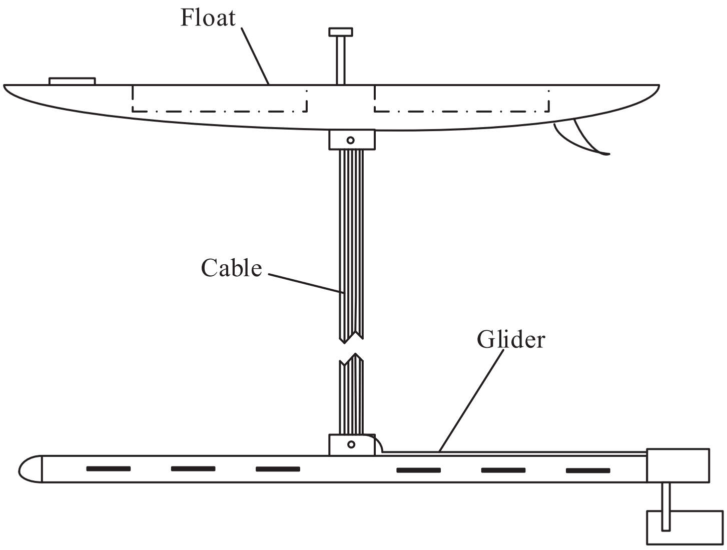

Figure S1. Conceptual diagram of the wave glider: It consists of float, cable, and glider.

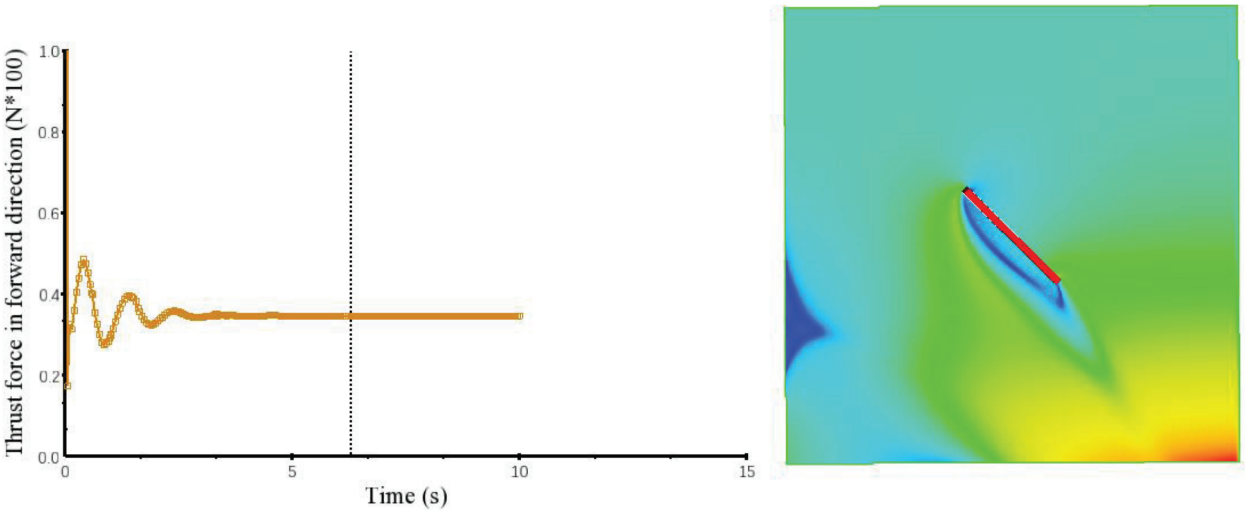

Figure S2. Example of one CFD simulation results: It can be seen that the thrust generated by the foil under the impact of water flow converges to the accurate value after a while.

Table S1. Sea trial record

	Date	Time	Wind	Wave	voyage
**Trad. Foil**	2021.05.10	$UTC\,3:08:39-3:37:58$	$\sim 3\,kt$	0.2-0.3 m	$190.59m$
**Asym. Foil**	2021.05.10	$UTC\,3:08:38-3:37:09$	$\sim 3\,kt$	0.2-0.3 m	$201.87m$

Table S2. Speed estimation of the wave glider

Trad. peak thrust	Speed-trad. foil	Speed-asym. foil	Speed enhancement
20 N	0.5462 m/s	0.5879 m/s	7.6%
15 N	0.4666 m/s	0.5022 m/s	7.6%
10 N	0.3845 m/s	0.4143 m/s	7.7%
5 N	0.2743 m/s	0.2920 m/s	7.7%

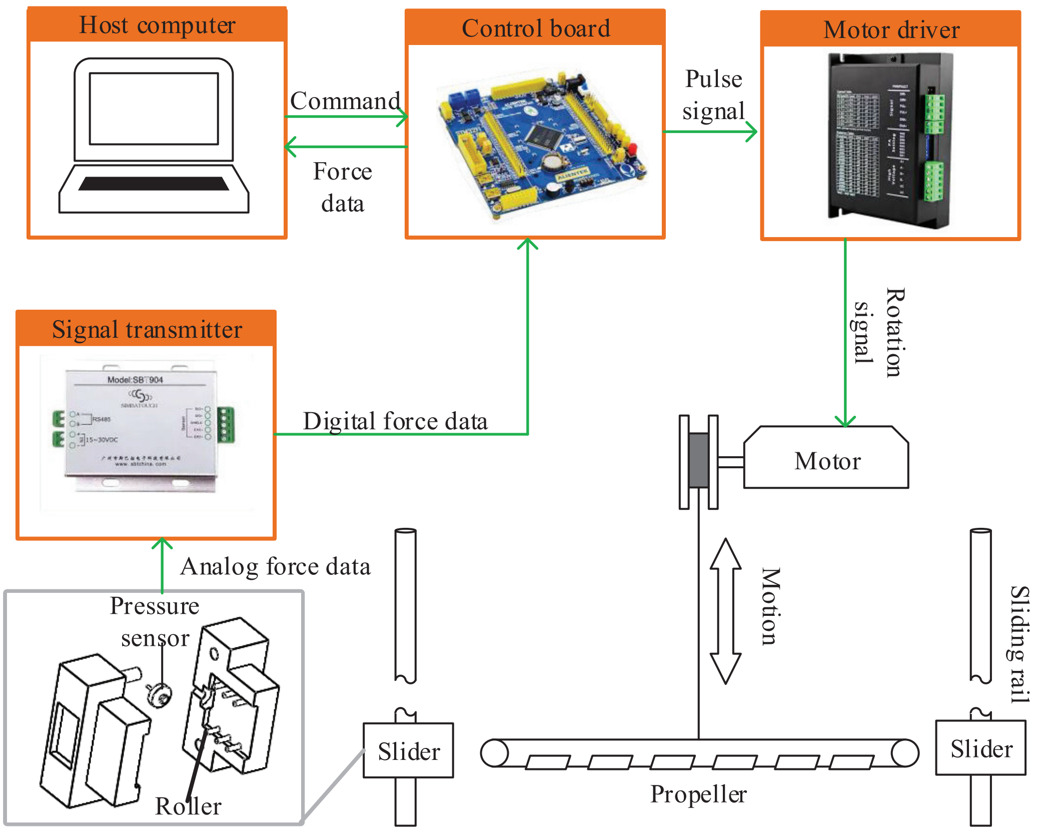

Figure S3. Experimental control system framework: a host computer, a control board, a motor drive, a motor , a pressure sensor, a signal transmitter and power source. In the experiment process, the motion command is sent to the control board through the host computer. After receiving the command, the control board outputs pulse signals with different step sizes to the motor driver according to the command's content. After receiving the pulse signal, the motor driver will rotate the motor with corresponding steps to achieve speed control. In the process of wave simulation, the output interval of each pulse is different to control the motor to produce different speeds.

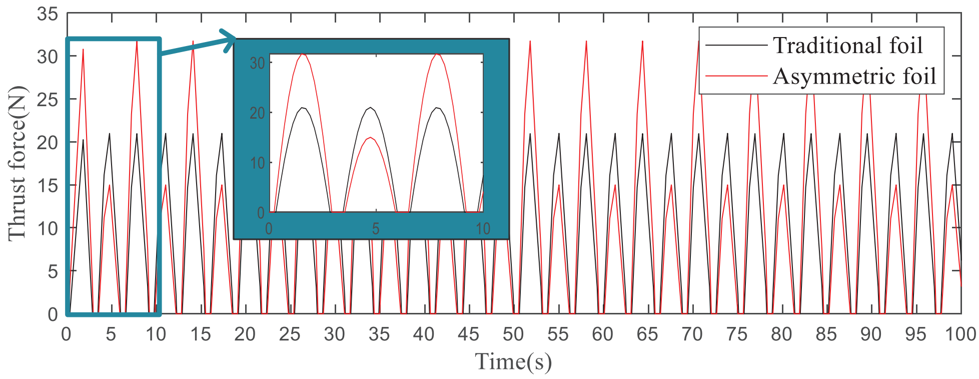

Figure S4. Thrust input of the wave glider's speed estimation. The different thrust produced by asymmetric foil and traditional foil are shown in the figure, obtained from the CFD cimulation.

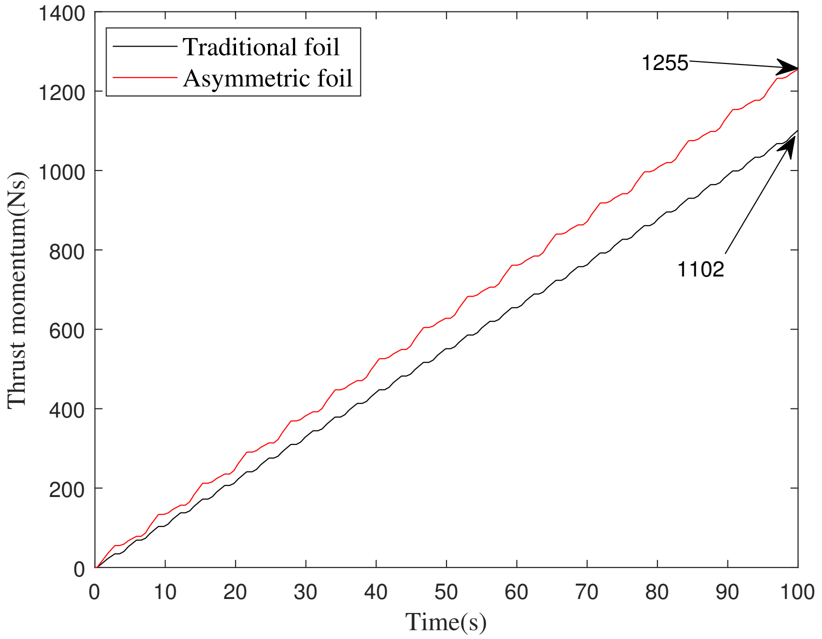

Figure S5. Thrust input of the numeric simulation: The different thrust produced momentum by asymmetric foil and traditional foil are shown in the figure. The total momentum enhancemnent is aslo 13.75%.

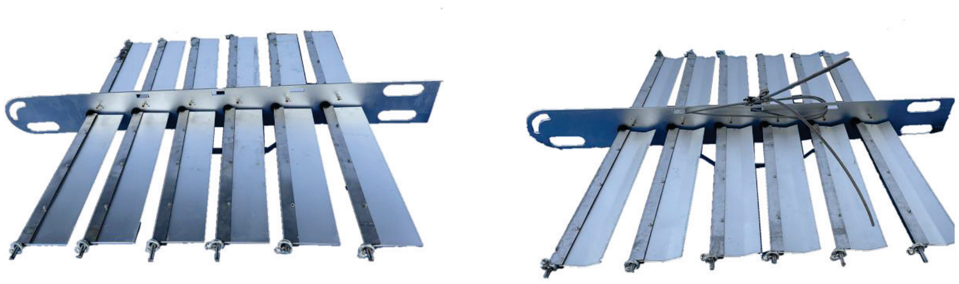

(a) (b)

Figure S6. Glider prototypes:(a) The glider with the traditional foils. (b) The glider with the asymmetric foils.

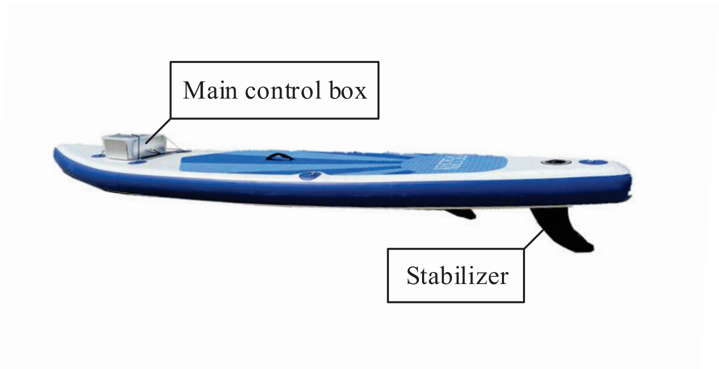

Figure S7. Float prototype: The main control box is equipped with Raspberry Pi with the GPS module and the IMU module. The stabilizer is used to reduce the rolling phenomenon of float.

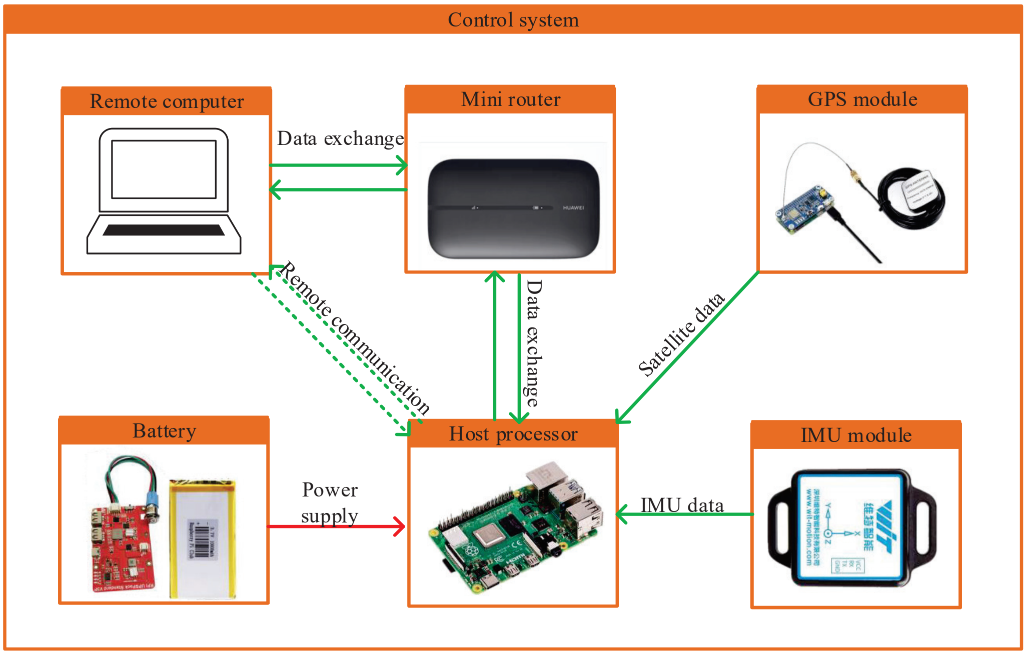

Figure S8. The control system of the prototype: The host processor is a *Raspberry Pi* board; Powered by a battery; The data collected by GPS module and IMU module is transmitted to the host processor; The host processor and remote computer are connected to a mini router to enter the LAN and communicate. GPS module will send multiple sets of data ($GPGGA, $GNGGA, $GPRMC, $GNRMC, ...), all following NEMA 0183 protocol. We only keep the sentence at the beginning of $GNRMC (multi-satellite integrated positioning data using GPS, BD2, and QZSS) and eliminate other data. The result of each location information is like "$GNRMC,045355.000,A,2234.4272,N,11432.7194,E,0.96,295.47,260321,,,A*7E". This sentence contains location information such as data header, sensor status, UTC, longitude and latitude, altitude, etc.

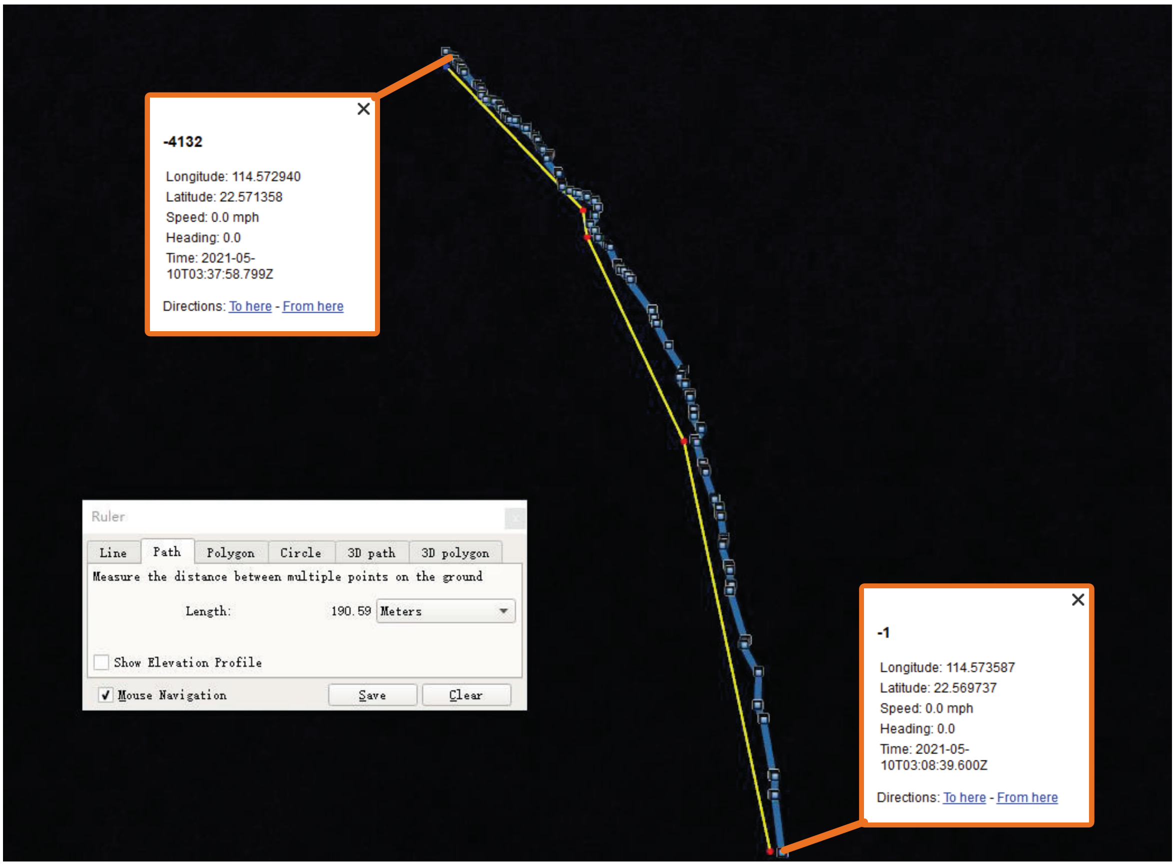

Figure S9. Sailing record of the wave glider with traditional foils: From 3:08:39 to 3:37:58; The sailing distance is 190.59 m.

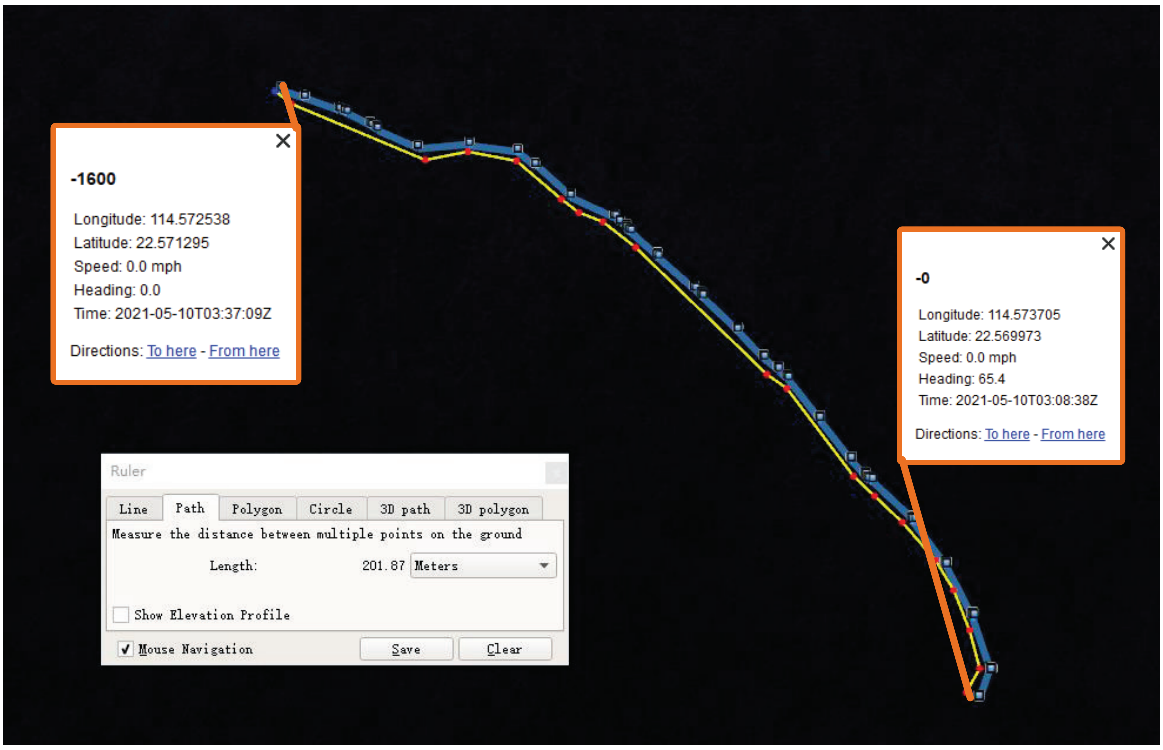

Figure S10. Sailing record of the wave glider with asymmetric foils: From 3:08:38 to 3:37:09; The sailing distance is 201.87 m.

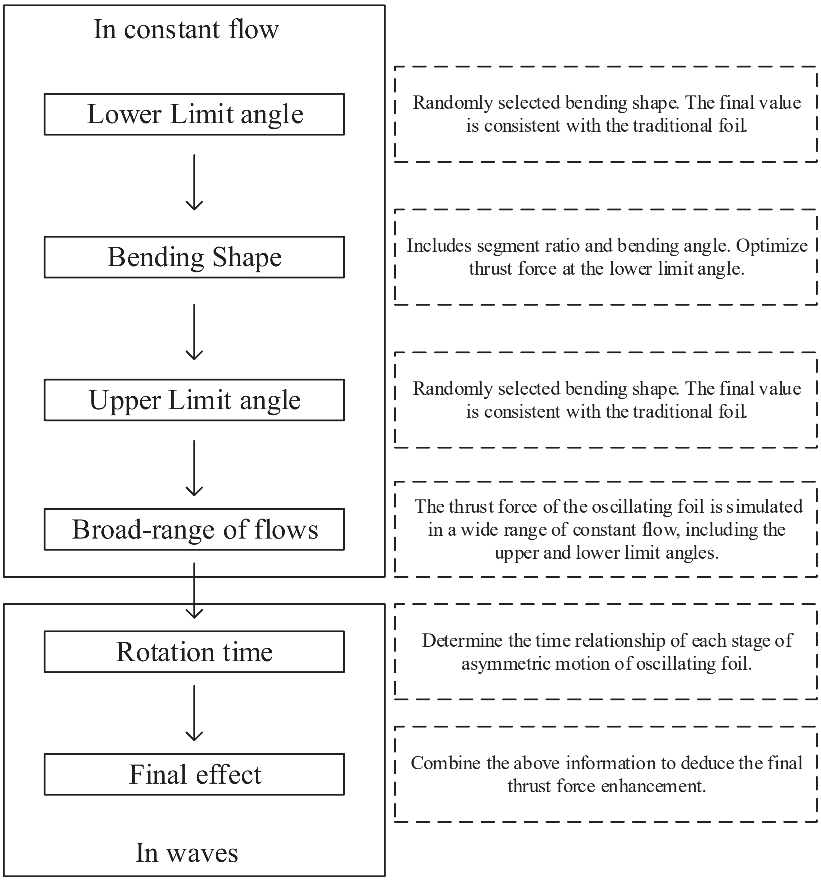

Figure S11. Key parameter establishment steps of the asymmetric oscillating foil..
